# *FAT-5/SCD5*-mediated lipid localization in lysosomes alleviates gamma radiation injury in *Caenorhabditis elegans*

**DOI:** 10.1016/j.jbc.2025.110674

**Published:** 2025-09-02

**Authors:** Tong Zhu, Zhouxuan Wang, Jiaze Li, Yu Zhao, Saijun Fan

**Affiliations:** Tianjin Key Laboratory of Radiation Medicine and Molecular Nuclear Medicine, Institute of Radiation Medicine, Chinese Academy of Medical Sciences and Peking Union Medical College, Tianjin, China

**Keywords:** radiation-induced damage, *GCY-5*, lysosomes, *FAT-5/SCD5*, lipid storage

## Abstract

Accidental internal or external exposure to gamma radiation can cause severe injury to the human body. The identification of an effective medication target has become particularly important for the treatment of radiation-induced injury. In this work, *Caenorhabditis elegans* was found to tolerate high-dose radiation when exposed to an extremely low-temperature environment (at 4 °C) for 4 h before irradiation. Experimental confirmation revealed that metabolites excreted by *C. elegans* targeted the guanylyl cyclase *GCY-5*. Furthermore, *GCY-5* activation by the excreted metabolites both suppressed lysosome acidification and promoted lipid translocation in lysosomes *via FAT-5/SCD5*. This ultimately resulted in the mitigation of radiation-induced damage. The pathway discovered in *C. elegans* was also confirmed in 293T cells, indicating a potential new target for radiation-induced damage research. A radiotherapy prognosis analysis based on the TCGA database indicated that assessing SCD5 expression in patients with kidney renal clear cell carcinoma (KIRC) during clinical treatment may aid in evaluating their suitability for radiotherapy. This study provides clinicians with a valuable basis for developing more effective therapeutic strategies and an experimental basis for future investigations in this area.

Ionizing radiation (IR) is commonly used in multiple industries, including nuclear power plants, medical treatment, food preservation, and product sterilization. Accidental and nonspecific exposure is accompanied by the application of IR (1, 2). Radiation-induced injury affects mainly the blood, digestive, and nervous systems (3, 4). The severity of the damage is directly related to the amount of radiation exposure. Injuries significantly impact the quality of life of patients and can even pose life-threatening risks.

It is believed that animals live longer at lower temperatures. Studies have shown that lower temperatures extend the lifespan of *Caenorhabditis elegans*. To date, the extended lifespan of *C. elegans* at low temperatures has been shown to be regulated by senescence-related pathways, such as the *DAF-16/FOXO*-mediated insulin/*IGF-1* signaling (IIS) pathway (5). Desaturase is essential for longevity in *C. elegans*. *MDT-15* and the adiponectin receptor *PAQR-2* increase the expression of the fatty acid desaturase *FAT-7*, which induces autophagy at low temperatures and promotes longevity (6). Several studies have shown that the maintenance of unsaturated fatty acids is the major cause of lifespan extension in *C. elegans* (7, 8, 9, 10).

In addition, the lysosomal pathway could be another important factor that influences the lifespan of *C. elegans*. Impaired lysosomal function affects the clearance of aggregation-prone proteins and affects the longevity of *DAF-2*, *EAT-2* and *ISP-1* mutants (11). An increase in lysosomal activity promotes the removal of toxic proteins and prolongs the lifespan of *C. elegans* (12). Furthermore, lysosomal function has implications for fatty acid desaturation. Lysosomes prolong lifespan by activating the nuclear hormone receptors *NHR-49* and *NHR-80* (13). *NHR-80* targets the fatty acid desaturase *FAT-6*, which promotes longevity by desaturating stearic acid to produce oleic acid (14). However, the impact of the interplay between lysosomal function and fatty acid metabolism on lifespan remains unclear.

In this work, the lifespan of *C. elegans* after irradiation was extended by exposing the organisms to 4 °C or treating them with medium that was excreted by *C. elegans* at 4 °C. After experimental confirmation, we showed that crosstalk between the lysosomal pathway and *FAT-5/SCD5*-mediated fatty acid desaturation was involved in the protective effect, which was regulated by the guanylyl cyclase *GCY-5* in ASER chemosensory neurons. Subsequent data analysis using patient samples from the TCGA database and experimental validation in the 293T cells identified the *SCD5*-mediated pathway as a key mechanism in the mitigation of radiation-induced damage. This work provides a new target for radiation-induced injury research and identifies a potential research direction for the development of treatments for radiation-induced injury.

## Results

### Extraction medium from *C. elegans* incubated at 4 °C mitigates radiation-induced injury

**Figure 1:**
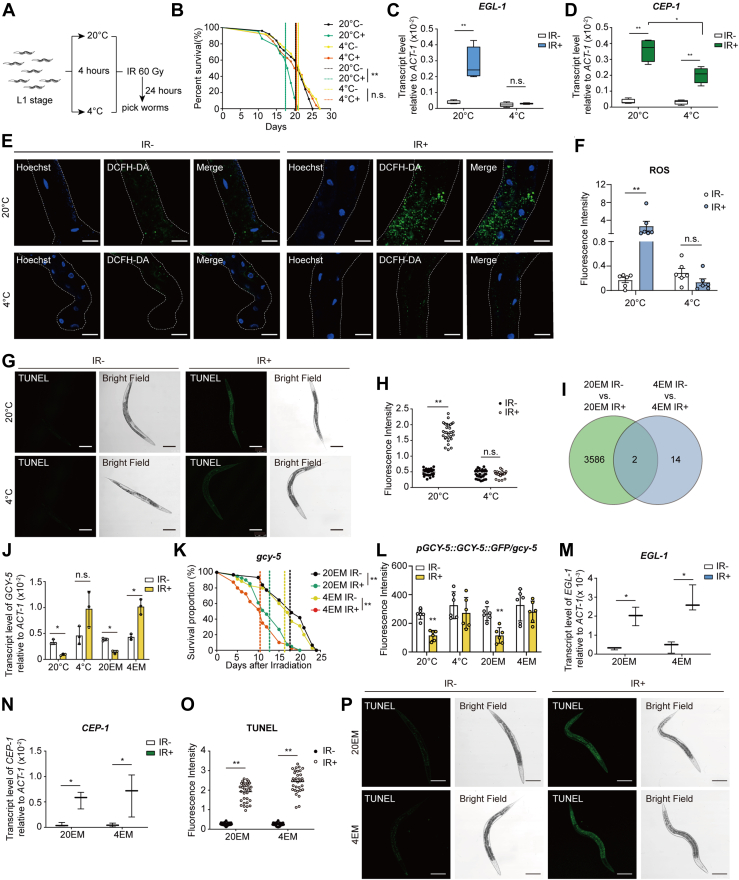
**Treatment at 4 °C alleviates radiation-induced injury in *C. elegans,* and *GCY-5* is involved in this biological process.***A*, experimental scheme of treatments at different temperatures. Worms in the L1 stage were exposed to 20 °C or 4 °C for 4 h. After 24 h, the worms were selected for further confirmatory experiments. *B*, Kaplan–Meier survival curves of *C. elegans* under different treatment conditions. “−” indicates the group that was not exposed to irradiation. “+” indicates the group that was exposed to irradiation. The *dotted lines* indicate the median lifespan of each group. ∗, *p* < 0.05; ∗∗, *p* < 0.01; n.s., not significant. *C* and *D*, the relative transcript levels of *EGL-1* (*C*) and *CEP-1* (*D*) in the different groups. At least three replicates were included for each group. For each plot, more than 1000 worms were collected for total RNA extraction. *ACT-1* was used as the reference gene. *E*, ROS accumulation in the intestines of worms. Scale bar, 25 μm. *F*, fluorescence intensity of the data in (*E*). For each group, more than six images were captured and analyzed. Individual data points for worms from each group are shown in the graph. *G*, images of TUNEL staining in the different groups, including WT nematodes stained with TUNEL staining solution. Scale bar, 100 μm. *H*, statistical analysis of the data in (*G*). For each group, over 30 images were captured and analyzed. Individual data points for worms from each group are shown in the graph. *I*, venn diagram of the differentially expressed genes in the 20EM and 4EM groups. The *green* area indicates the genes whose expression significantly differed in the 20EM group after irradiation. The *blue* area indicates the genes whose expression significantly differed in the 4EM group after irradiation. Two genes are in both of these regions. *J*, transcription level of *GCY-5* after exposure to different temperatures or EM. At least three replicates were included for each group. ∗, *p* < 0.05; ∗∗, *p* < 0.01; n.s., not significant. *K*, Kaplan–Meier survival curve analysis of the *GCY-5*-deficient line after administration of 20EM and 4EM treatment with or without irradiation. ∗, *p* < 0.05; ∗∗, *p* < 0.01; n.s., not significant. *L*, fluorescence intensity of *pGCY-5::GCY-5::GFP/gcy-5* worms in different groups as read by a microplate reader. There were 50 worms in each well. The vertical ordinate represents the fluorescence intensity of 10 worms in different groups. At least three replicates were included in each group. *M* and *N*, relative transcript levels of *EGL-1* (*M*) and *CEP-1* (*N*) in *gcy-5*-deficient worms after administration of 20EM or 4EM treatment with or without irradiation. At least three replicates were included for each group. *ACT-1* was used as the reference gene. ∗, *p* < 0.05; ∗∗, *p* < 0.01; n.s., not significant. *O* and *P*, images of TUNEL staining of *gcy-5*-deficient worms treated with 20EM or 4EM with or without irradiation (*P*) and statistical analysis of the images (*O*). Scale bar, 100 μm.

To investigate the effect of temperature on radiation-induced injury in *C. elegans*, we performed several irradiation assays at 20 °C, 15 °C, 10 °C, 4 °C, and 0 °C (Fig. S1, *A*–*C*) following the scheme shown in Figure 1A. Unexpectedly, we found that irradiated worms had a more normal lifespan when treated at 4 °C (or lower temperatures, Fig. S1*C*) than when treated at 20 °C (Fig. 1*B*). We further investigated the relative expression levels of four genes involved in apoptosis (15, 16). The transcript levels of *EGL-1*, *CEP-1*, *CED-3,* and *CED-4* in worms treated at 20 °C were increased after exposure to gamma radiation, which indicated more severe injury. However, the transcript levels of these four genes were maintained at normal levels in worms subjected to short-term incubation at 4 °C before irradiation (Figs. 1, *C* and *D*, and S1, *D*–*E*). In addition, the accumulation of reactive oxygen species (ROS) induced by irradiation was significantly decreased after short-term incubation at 4 °C (Fig. 1, *E* and *F*). Furthermore, we performed TUNEL staining to visualize apoptosis in worms. Consistent with the aforementioned data, short-term incubation at 4 °C decreased the fluorescence intensity in worms that were stained after irradiation at 60 Gy (Fig.1, *G* and *H*). These results confirmed that short-term incubation at 4 °C mitigated the injury induced by gamma radiation.

To improve clinical treatment strategies, we sought to identify a method that remains effective independent of low-temperature conditions. Since *C. elegans* might secrete gut-based microorganisms into the medium, we further performed another experiment to confirm the influence of flora in the medium. After the quantification of the mRNA levels of *EGL-1*, *CEP-1*, *CED-3* and *CED-4*, we excluded the effects of gut-based microorganisms released into the environment (Fig. S1, *F*–*G*). Thus, we purified the medium with filters and centrifugation following the scheme shown in Figure S1*H*. Untreated worms were incubated in the extract medium (EM) at 20 °C and exposed to gamma radiation at a dose of 60 Gy. The median lifespan in the 4EM group was significantly longer than that in the 20EM group after irradiation (Fig. S1*I*). Similarly, 4EM treatment maintained the relative expression of *EGL-1*, *CEP-1*, *CED-3*, and *CED-4* at normal levels after irradiation, whereas the expression of these genes was increased in the 20EM group (Fig. S1, *J*–*M*). TUNEL staining confirmed these findings, as the fluorescence intensity was significantly increased in 20EM-treated worms after irradiation. There was no change in fluorescence intensity in 4EM-treated worms (Fig. S1, *N* and *O*). Furthermore, ROS accumulation in the intestines decreased after 4EM treatment (Fig. S1, *P* and *Q*). These data indicate that 4EM could directly mitigate radiation-induced injury in *C. elegans* in the absence of a change in temperature.

### The pathway mediated by the guanylyl cyclase *GCY-5* is involved in the protective effect of 4 °C

To study the metabolic processes associated with the protective effect of 4EM, we conducted transcriptome analysis of worms treated with 20EM or 4EM and irradiated at 60 Gy or not. The genes that were differentially expressed between the 20EM IR− and the 20EM IR+ groups and between the 4EM IR− and 4EM IR+ groups are shown in a Venn diagram (Fig. 1*I*). The comparison between the 4EM IR− and 4EM IR+ groups revealed only 16 differentially expressed genes, whereas the comparison between the 20EM IR− and 20EM IR+ groups identified 3588 differentially expressed genes. These results indicated that the changes in gene expression induced by irradiation following 20EM treatment and those following 4EM treatment were completely different. To identify specific target genes of 4EM, we analyzed 14 genes whose expression was specifically induced by 4EM. *GCY-5* is a guanylyl cyclase in *C. elegans* that is expressed in chemosensory neurons. Given its emergence within this group and its involvement in the regulation of the superoxide dismutase *SOD-1* (17), we investigated the transcriptional levels of *GCY-5* in *C. elegans*. The level of *GCY-5* in 20EM-treated worms and worms incubated at 20 °C was significantly decreased following irradiation (Fig. 1*J*). However, it was elevated or unchanged following treatment with 4EM. Subsequent observations were conducted to assess the lifespan of mutant worms subjected to irradiation and 4EM treatment in the absence of *GCY-5*. The findings indicated that irradiation reduces the lifespan of the *gcy-5-deficient* worms (*RB1000*); however, the 4EM treatment proved to be ineffective in the absence of *GCY-5* (Fig. 1*K*). In addition, a transgenic line, *pGCY-5::GCY-5::GFP/gcy-5,* based on *gcy-5*-deficient worms (*PHX4456*), was generated to investigate the expression and function of *GCY-5*. As the fluorescence intensity of *pGCY-5::GCY-5::GFP/gcy-5* was elevated both after 4 °C and 4EM treatment, as shown by data from a microplate reader (Fig. 1*L*). The fluorescence intensity observed *via* confocal microscopy in the overexpressed nematodes demonstrated consistent results (Fig. S2*A*). We further investigated the relative expression of *EGL-1*, *CEP-1*, *CED-3,* and *CED-4* in *gcy-5*-deficient worms after irradiation. 4EM treatment did not reduce the relative expression of *EGL-1*, *CEP-1 CED-3* or *CED-4* as it did in *gcy-5* worms after irradiation (Figs. 1, *M* and *N* and S2, *B* and *C*). For further confirmation, TUNEL staining was performed in *gcy-5*-deficient worms to visualize radiation-induced damage. The fluorescence intensity was significantly increased in both 20EM-treated worms and 4EM-treated worms after irradiation (Fig. 1, *O* and *P*). These data indicate that *GCY-5* is a key gene involved in 4EM-mediated protection against radiation.

### 4EM suppresses the acidification of lysosomes through *GCY-5*

**Figure 2:**
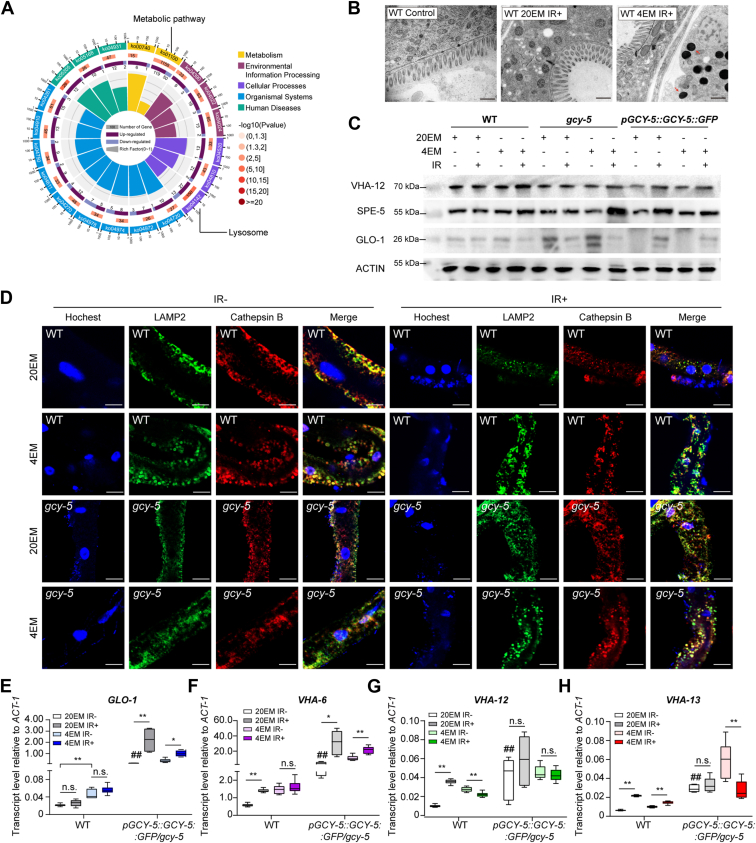
**Lysosomal function is affected by *GCY-5*.***A*, circular map of Kyoto Encyclopedia of Genes and Genomes (KEGG) analysis of the genes between the *GCY-5* 4EM IR+ and WT 4EM IR+ groups. The different colors shown in the outer circle represent different KEGG classes. The numbers within the middle circle indicate the quantity of differentially expressed genes within each pathway. The background color of these numbers corresponds to the *p* value of the differentially expressed genes in the respective pathway, as indicated in the *rightpanel*. *B*, TEM images of different groups. The *red arrowhead* indicates lysosomes. Scale bar, 500 nm. *C*, Western blotting of VHA-12, SPE-5 and GLO-1 expression in WT, *gcy-5* mutant, and *pGCY-5::GCY-5::GFP/gcy-5* worms. *D*, images of lysosome fluorescence staining in the intestines of WT and *gcy-5*-deficient worms subjected to different treatments. The images in the first two rows are of WT worms. The images in the *bottom* two rows are of *gcy-5* worms. Lysosomes were stained with LAMP2 (GFP). Acidic lysosomes were stained with cathepsin B (RFP). Nuclei were stained with Hoechst. Scale bar, 10 μm. *E–H*, relative transcript levels of *GLO-1* (*E*), *VHA-6* (*F*), *VHA-12* (*G*), and *VHA-13* (*H*) in the WT (*left*) and *pGCY-5::GCY-5::GFP/gcy-5* (*right*) strains after 20EM or 4EM treatment with or without irradiation. At least three replicates were included for each group. *ACT-1* was used as the reference gene. ∗, *p* < 0.05, ∗∗, *p* < 0.01; n.s., not significant. #, *p* < 0.05 compared with WT 20EM IR-.

To investigate the mechanism underlying the protective effect of *GCY-5* against radiation-induced injury, we performed transcriptome analysis of *gcy-5*-deficient and wild-type (WT) worms treated with 4EM. More than 3000 genes were significantly differentially expressed after irradiation in *gcy-5*-deficient worms treated with 4EM compared with WT worms treated with 4EM (Fig. S2*D*). The enriched pathways are shown in Figure 2*A*. Most of the differentially expressed genes were enriched in the metabolic pathways (1158 genes) or lysosomal pathways (115 genes). Additionally, the lysosomal pathway emerged as the most significant pathway based on the *p* values of differentially expressed genes (Figs. 2*A* and S2*E*).

We further assessed the morphology of lysosomes and lysosomal function in *C. elegans* after irradiation. Transmission electron microscopy (TEM) revealed that the number of lysosomes was unaltered after irradiation or 4EM treatment. However, 4EM treatment resulted in increased production of lysosomes and increased accumulation of substrates within them (Fig. 2*B*). We further investigated the expression of proteins involved in lysosomal function. Western blot analysis revealed that the expression of GLO-1, a protein associated with lipid storage, was not changed significantly in WT. In contrast, it was significantly increased in *gcy-5-deficient* lines under normal conditions (20EM IR-). In gcy-5-deficient lines, GLO-1 was affected by irradiation significantly. Conversely, in the *pGCY-5::GCY-5::GFP/gcy-5* line, GLO-1 levels were reduced under normal conditions but significantly increased following irradiation, independent of 4EM treatment (Figs. 2*C* and S2*H*). VHA-12 and SPE-5 are involved in the construction and acidification of lysosomes. VHA-12 and SPE-5 accumulated after 4EM IR+ both in WT and *gcy-5-*deficient worms, whereas they remained unchanged under other treatments (Figs. 2*C* and S2, *F*–*G*). This finding suggests that the acidification of lysosomes was not significantly affected by other treatments but 4EM IR+. However, the overexpression of GCY-5 resulted in contrasting outcomes with respect to acidification (Figs. 2*C* and S2, *F*–*H*). These results suggest that *GCY-5* is likely related to lipid storage under irradiation and that it suppresses the acidification of lysosomes. To further investigate the relationship between *GCY-5* and lysosomal function after irradiation, we stained the worms with cathepsin B to evaluate lysosomal acidification and LAMP2 to evaluate the structure of the lysosomes. Gamma radiation did not affect the colocalization of LAMP2 and cathepsin B, indicating that lysosomal function was not affected by irradiation or was even promoted (not assessed). However, 4EM significantly reduced the degree of colocalization in WT worms independent of irradiation (Fig. 2*D* and S2*I*). On the other hand, lysosomal function was not influenced by irradiation or EM treatment in *gcy-5*-deficient worms (Fig. 2*D* and S2*I*). *VHA-12* and *VHA-13* are essential for lysosomal lumen acidification (18, 19). *GLO-1* affects the formation of lysosome-related organelles (20) and is involved in epithelial polarization through the lysosome pathway in the intestines (21). VHA-6 is in lysosomes and is responsible for luminal acidification and fat storage (22). For further confirmation, the expression of *GLO-1*, *VHA-6*, *VHA-12*, and *VHA-13* was detected (Fig. 2, *E–H*). The WT worm data revealed that the formation and acidification of lysosomes were significantly increased after irradiation due to the upregulation of *VHA-12* and *VHA-13* (Fig. 2, *G* and *H*). Moreover, the expression levels of *GLO-1* and *VHA-6* were elevated after 4EM treatment (Fig. 2, *E* and *F*). These results indicate that the lysosomal pathway might be promoted by gamma radiation. However, 4EM suppressed the function of lysosomes and promoted the *GLO-1* and *VHA-6*-mediated organization of fat storage. In the *pGCY-5::GCY-5::GFP/gcy-5* line, both the function of lysosomes and the degree of lipid storage were increased after irradiation or EM treatment (Fig. 2, *E*–*H*). Thus, irradiation and EM treatment altered different aspects of lysosomal function, including fat storage, protein transportation, and acidification through *GCY-5*.

### Alterations in lipid distribution are promoted by the *GCY-5*-mediated pathway after irradiation

**Figure 3:**
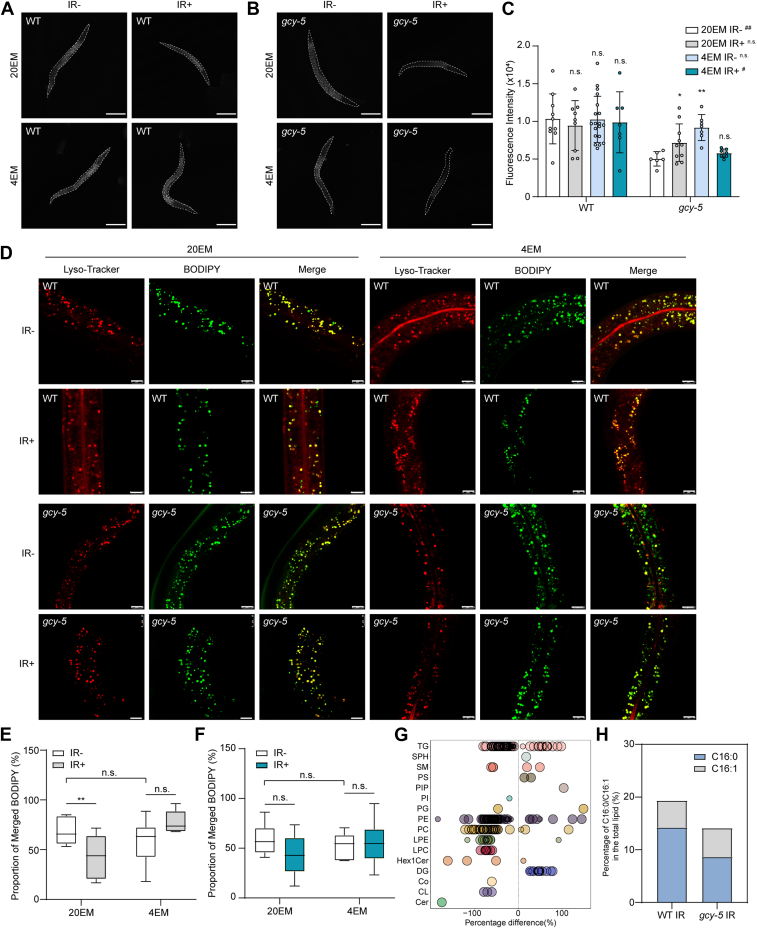
**Lipid distribution is affected by *GCY-5*.***A-B*, *Nile red* staining of whole WT (*A*) and *gcy-5* (*B*) worms. Images show raw data. Scale bar, 100 μm. (*C*) Statistical analysis of the data in (*A*) and (*B*). *D*, images of BODIPY and LysoTracker staining in WT and *gcy-5* worms. *Green* particles indicate stained lipids. *Red* particles indicate stained lysosomes. Scale bar, 25 μm. *E* and *F*, statistical analysis of the data in (*D*), including WT (*E*) and *gcy-5* (*F*) worms. The merged BODIPY-stained particles in each image were counted *via* ImageJ software. For each group, more than six images were captured and analyzed. Individual data points for worms from each group are shown in the graph. ∗∗, *p* < 0.01; n.s., not significant. *G*, Bubble plot of changed lipids in the lipidomic analysis. *H*, ratio of C16:0/C16:1 in the lipidomic analysis between the WT IR- and gcy-5 IR-groups.

Considering the transcriptome results, a significant proportion of differentially expressed genes showed enrichment in metabolic pathways. Further annotation of the metabolic pathways revealed that lipid metabolism was the most prominently affected pathway (Fig. S3*A*). Thus, we investigated the total amount of fat in the worms *via* Nile red staining. The results revealed that irradiation did not affect fat storage in wild-type worms, whereas fat storage was reduced in *gcy-5*-deficient worms even without irradiation (Fig. 3, *A*–*C*). As a result, irradiation increased the fat storage in *gcy-5* worms, whereas subsequent treatment with 4EM decreased fat storage levels to those observed in the 20EM IR-group (Fig. 3, *A*–*C*). These results suggest the direct involvement of both 4EM and *GCY-5* in the regulation of fat storage processes.

To determine the ratio of lipids in lysosomes, we stained intestines isolated from worms with BODIPY and LysoTracker. Lipids in lysosomes were significantly decreased in wild-type worms after irradiation, whereas 4EM reversed this decrease (Fig. 3, *D* and *E*). The ratio of merged lipids was not influenced by irradiation in *gcy-5-*deficient worms (Fig. 3, *D* and *F*). Compared with that of wild-type worms in the 20EM IR-group, the ratio of merged lipids in *gcy-5* mutants did not change, which means that *GCY-5* was involved only in gamma radiation-induced alterations in lipid distribution (Fig. 3, *D*–*F*). To explore the mechanism underlying fatty acid metabolism related to *GCY-5*, we analyzed the transcriptome results shown in Figures 2*A* and S3*A* combined with the map of the fatty acid metabolism pathway. Figure S3*B* shows the fatty acid metabolism pathway (KEGG ID: cel01212) and indicates genes that were differentially expressed after *GCY-5* deletion. Most of the differentially expressed genes involved in this pathway were upregulated, whereas only *FAT-5*, located at the critical node of the whole pathway, was downregulated (Fig. S3*B*). To further validate our findings, we conducted a nontargeted lipidomic analysis of WT IR+ and gcy-5 IR+ samples to identify key genes located downstream of GCY-5. Our analysis revealed significant alterations in several lipid types, including triglycerides (TGs), which are influenced primarily by FAT-5, following the deletion of GCY-5 (Fig. 3*G*). FAT-5 has been identified as a crucial regulator in the process of C16:0 desaturation to C16:1 (23). Consequently, we quantified the levels of C16:0 and C16:1 in both groups. The results indicated that a deficiency in GCY-5 leads to a reduction in the total amount of C16. Furthermore, the C16:0/C16:1 ratio decreased from 2.79 to 1.57 (Fig. 3*H*). This result suggests that *FAT-5* might be the key downstream gene regulated by *GCY-5*.

### The *FAT-5*-mediated pathway affects lysosomal function under gamma radiation conditions

**Figure 4:**
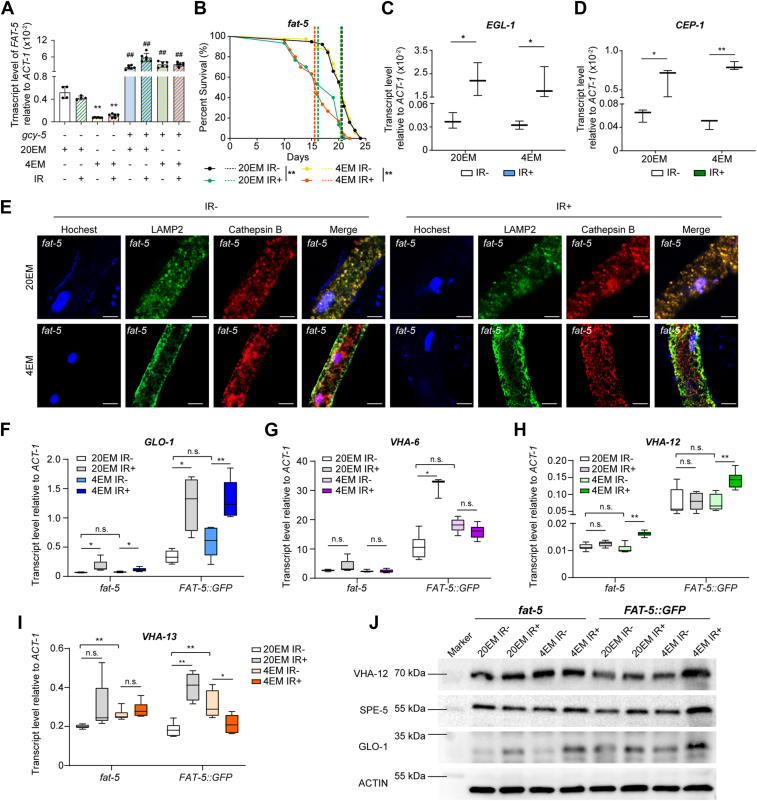
***FAT-5* is involved in the fatty acid metabolism-related lysosomal pathway.***A*, transcript levels of *FAT-5* in different lines under different treatment conditions. The *left* four bars show the results from the *gcy-5* mutant lines. The *right* four bars show the results from the WT line. At least three replicates were included for each group. More than 1000 worms were collected from each sample. ∗, *p* < 0.05; ∗∗, *p* < 0.01; n.s., not significant. ##, *p* < 0.01, compared with the gcy-5 mutant line. *B*, Kaplan–Meier survival curve analysis of *fat-5* worms after 20EM or 4EM treatment with or without irradiation. The dotted lines indicate the average lifespan of each group. ∗∗, *p* < 0.01. *C*–*D*, relative transcript levels of *EGL-1* (*C*) and *CEP-1* (*D*) in *fat-5* worms after 20EM or 4EM treatment with or without irradiation. At least three replicates were included for each group. *ACT-1* was used as the reference gene. ∗, *p* < 0.05, ∗∗, *p* < 0.01; n.s., not significant. *E*, images of fluorescence staining of lysosomes with LAMP2 and cathepsin B in the intestines of *fat-5*-deficient worms subjected to different treatments. Nuclei were stained with Hoechst. Scale bar, 10 μm. *F–I*, fold change in the expression of *GLO-1* (*F*), *VHA-6* (*G*), *VHA-12* (*H*), and *VHA-13* (*I*) after 4EM treatment in *fat-5* and *fat-5::GFP C. elegans*. At least three replicates were included for each group. More than 1000 worms were collected from each sample. ∗, *p* < 0.05; ∗∗, *p* < 0.01; n.s., not significant. *J*, Western blotting to detect VHA-12, SPE-5 and GLO-1 expression in *fat-5* and *fat-5::GFP* worms.

Fatty acid metabolism is highly relevant to lifespan (24). Before we performed subsequent experiments, we assessed the transcription level of *FAT-5* in wild-type worms treated with 20EM or 4EM after irradiation. Although 4EM reduced the transcription level of *FAT-5* after treatment with 4EM, irradiation did not significantly influence the expression of *FAT-5* after either 20EM or 4EM treatment (Fig. 4A). On the other hand, *gcy-5* deficiency led to significant downregulation of *FAT-5* expression after 4EM treatment in both nonirradiated and irradiated worms (Fig. 4*A*). These results show that 4EM and gamma radiation are not key factors regulating *FAT-5* expression. Moreover, *GCY-5* increases the transcription level of *FAT-5* independent of exposure to gamma radiation. To validate these results, we further evaluated the lifespan of *fat-5* mutant worms treated with 20EM or 4EM after irradiation. The average lifespan of *fat-5* worms treated with 20EM after irradiation was significantly reduced to 16 days (Fig. 4*B*). In addition, 4EM did not reverse the reduction in the average lifespan induced by irradiation in the *fat-5* mutant line (Fig. 4*B*). Furthermore, the expression of related response genes was significantly upregulated after irradiation, even after treatment with 4EM, in the *fat-5* mutant line (Fig. 4, *C* and *D*). These results suggest that *FAT-5* functions downstream of *GCY-5*.

Since *FAT-5* and the lysosomal pathway are involved in the alleviation of radiation-induced damage in *C. elegans*, we speculated that there is a relationship between *FAT-5* and the lysosomal pathway. We first assessed whether lysosomal function was influenced by *FAT-5*. After staining with LAMP2 and cathepsin B, we found that lysosomal function was promoted after irradaition in the *fat-5*-deficient line, which is consistent with the results obtained in WT worms. However, 4EM did not affect the acidification of lysosomes in the absence of FAT-5 (Figs. 4*E* and S4*A*). Real-time PCR of *FAT-5*-deficient and *FAT-5*-overexpressing worms was carried out to measure changes in the expression of genes involved in the lysosomal pathway. According to the results shown in Figure 4, *F*–*I*, the expression of *VHA-6*, *VHA-12* and *VHA-13* was not affected by irradiation or 4EM treatment in the *fat-5* line. However, *GLO-1* was significantly upregulated by irradiation under both 20EM and 4EM treatment in *fat-5* worms (Fig. 4*F*), whereas was not changed in WT worms (Fig. 2*E*). These findings suggest that GLO-1 might mediate a lipid storage pathway under irradiation that is independent of FAT-5. In the *FAT-5::GFP* lines, the mRNA levels of all the genes were elevated compared with those in the *fat-5* lines. Moreover, the level of each gene was highly sensitive to irradiation in the *FAT-5::GFP* line (Figure 4F4I). These results were confirmed by protein levels determined *via* Western blotting. GLO-1 accumulated in response to irradiation in the *fat-5* line, especially after 4EM treatment (Fig. 4*J*). SPE-5 accumulation remained unchanged after irradiation in *fat-5-deficient* worms under both 20EM and 4EM (Figs. 4*J* and S4*F*). In the *FAT-5::GFP* line, the amount of GLO-1 was generally high relative to that in the *fat-5-deficient* line. All three proteins were promoted by irradiation in the *FAT-5::GFP* line, especially after 4EM treatment. These results suggest that *FAT-5* is involved in the fatty acid metabolism-related lysosomal pathway after irradiation and might be distinct from the pathway induced by GLO-1.

**Figure 5:**
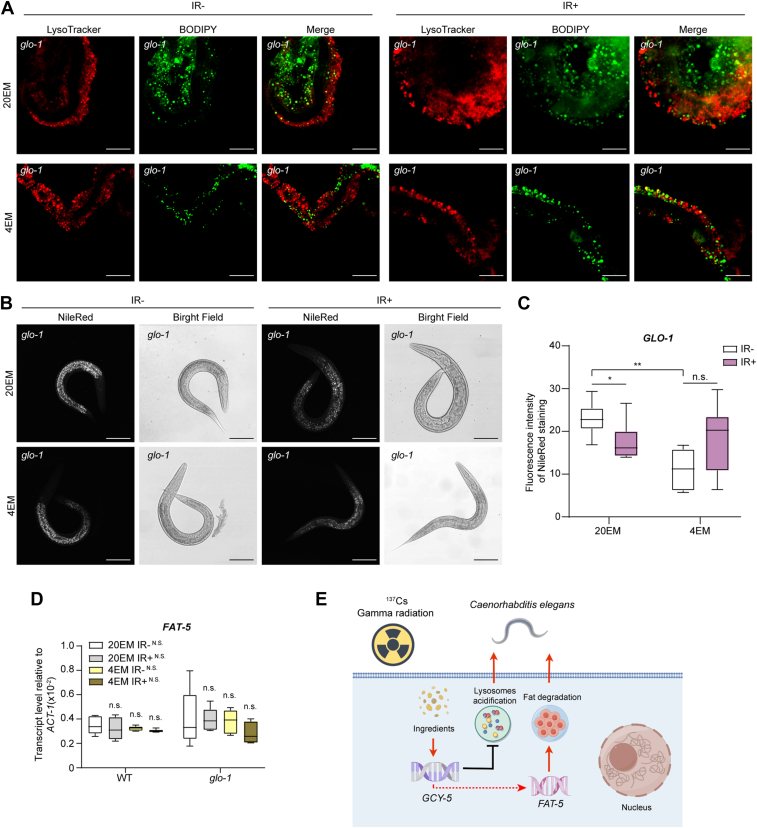
***Fat-5* regulates lipid distribution in lysosomes in a unidirectional manner.***A*, Images of BODIPY- and LysoTracker-stained *glo-1*-deficient worms. *Green* particles indicate the stained lipids. *Red* particles indicate stained lysosomes. Scale bar, 25 μm. *B*, *nile red* staining of whole *glo-1-*deficient worms. *C*, statistical analysis of the data in (*B*). For each group, more than six images were captured and analyzed. ∗, *p* < 0.05; ∗∗, *p* < 0.01; n.s., not significant. *D*, transcription level of *FAT-5* in WT and *glo-1-*deficient *C. elegans*. At least three replicates were included for each group. More than 1000 worms were collected from each sample. n.s., not significant, compared with 20EM IR-with the same line. N.S., not significant difference compared with the same treatment in the WT line. *E*, schematic diagram of the mechanisms in this work. *Red lines* and *arrows* indicate promotion. *Black lines* indicate suppression.

To further identify the relationship between lysosomes and the *FAT-5*-mediated fatty acid metabolism pathway, we investigated the lipid distribution in the *glo-1* mutant lines *via* BODIPY and LysoTracker staining. Lipid storage in lysosomes was significantly decreased in *glo-1* mutant worms (Fig. 5*A*). Moreover, the total amount of lipids in *glo-1* mutant worms was decreased after irradiation. Compared with 20EM IR-, 4EM treatment did not increase the total amount of lipids in *glo-1* mutant worms even after irradiation (Fig. 5, *B* and *C*). Additionally, the mRNA level of *FAT-5* was not affected by *GLO-1* deletion, which means that the regulation of lipid distribution in lysosomes by *FAT-5* is independent of GLO-1 (Fig. 5*D*). The regulatory mechanisms are shown in the schematic diagram in Figure 5*E*.

### The homologous protein *SCD5* increases survival rates following radiotherapy in 293T cells *via* a similar pathway

To assess the potential clinical application of the pathway found in this work, we compared the expression of *SCD5* between adjacent and cancerous tissues in different tumor types based on the public TCGA database (Fig. S6*A*). Consistent with previous findings by Jukun Song *et al.* (25), a decrease in SCD5 significantly influenced the survival of only patients with KIRC (Fig. S6, *B*–*G*). Given the association of SCD5 with radiation-induced damage identified in this study, we conducted a further analysis of the survival outcomes of KIRC patients based on their SCD5 expression levels. The results indicated that patients with high SCD5 expression had increased survival following radiotherapy (Fig. S7, *A* and *B*).

**Figure 6:**
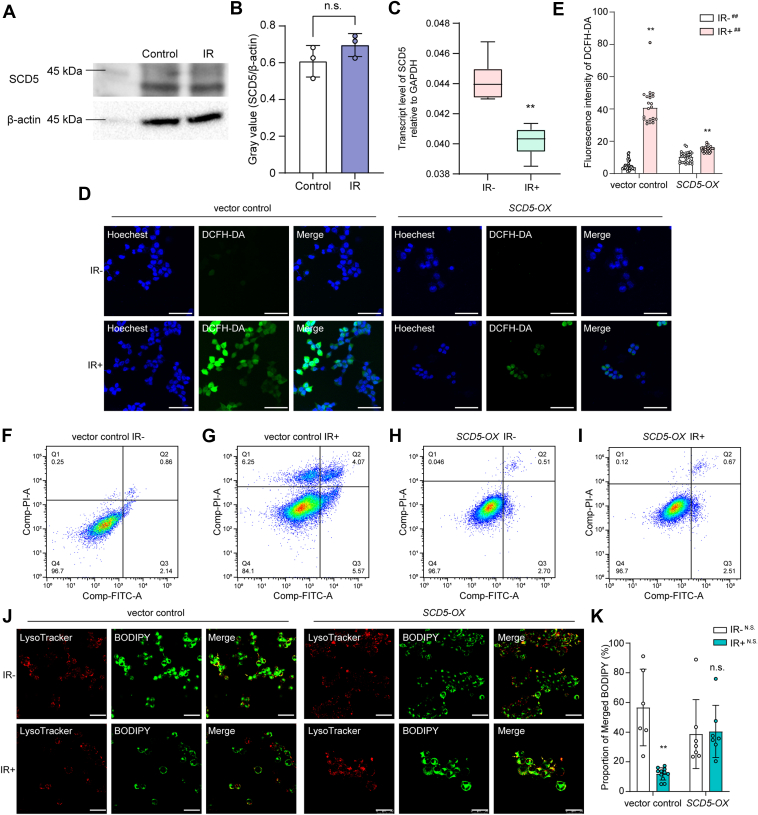
***SCD5* mitigates radiation-induced injury through a similar pathway in 293T cells.***A*, Western blotting to detect SCD5 expression in 293T cells with or without irradiation. *B*, Quantification of SCD5 protein levels. The error bars in graphs means standard deviation of indicated data. *C*, transcript level of *SCD5* in 293T cells with or without radiation exposure. At least three replicates were included for each group. ∗∗, *p* < 0.01. *D*, ROS accumulation in 293T cells after irradiation. Scale bar, 50 μm. *E*, fluorescence intensity of the data in (*D*). Individual data points for cells from each group are shown in the graph. ∗∗, *p* < 0.01 compared with IR-in each cell line; ##, *p* < 0.01 compared with cell lines subjected to various radiation treatments. *F–I*, annexin V-FITC/PI flow cytometry analysis of apoptotic cells in the vector control IR− (*F*), vector control IR+ (*G*), *SCD5-OX* IR− (*H*), and *SCD5-OX* IR+ (*I*) groups. *J*, images of BODIPY and LysoTracker staining in vector control and *SCD5-OX* cells. *Green* particles indicate stained lipids. *Red* particles indicate stained lysosomes. Scale bar, 50 μm. *K*, statistical analysis of the data in (*J*). The merged BODIPY-stained particles in each image were counted *via* ImageJ software. Individual data points for cells from each group are shown in the graph. ∗∗, *p* < 0.01; n.s., not significant.

Based on these results, we investigated the pathway induced by *SCD5* in 293T normal human renal epithelial cells. In 293T cells, the protein level did not change after irradiation (Fig. 6, *A* and *B*). However, mRNA level of *SCD5* decreased after irradiation (Fig. 6*C*). In this scenario, where the protein levels do not correspond with the mRNA levels, we hypothesize that this discrepancy may be attributed to alternative protein regulatory pathways. Consequently, we conducted additional experiments using 293T cells to ascertain whether the regulation of lipid storage by SCD5 in these cells aligns with the patterns observed in nematodes. To further validate this result, we generated a transgenic 293T cell line overexpression *SCD5 via* a modified pEGFP vector devoid of fluorescent protein (*SCD5-OX*). After exposure to irradiation, the accumulation of ROS in the *SCD5-OX* cell lines was significantly decreased (Fig. 6, *D* and *E*). In addition, the number of apoptotic cells after irradiation was also decreased compared with that in the vector control group, as shown by Annexin V-FITC/PI flow cytometry detection (Fig. 6, *F*–*I*). We further detected the lipid distribution in *SCD5-OX* cells. The localization of lipids within lysosomes was significantly reduced following irradiation in vector control cells. Conversely, lipid localization was increased upon the overexpression of *SCD5* (Fig. 6, *J* and *K*). These findings suggest that *SCD5* can regulate the translocation of lipids into lysosomes in human cell lines, in addition to its effects in *C. elegans*.

## Discussion

Gamma radiation induces multiple visceral injuries in humans. The clinical options for radiation-induced injury treatment are limited. Our work revealed a specific *SCD5*-mediated pathway after irradiation in both *C. elegans* and 293T cells, which could mitigate radiation-induced injury by promoting the lipid localization in lysosomes.

### *GCY-5* senses specific components and differs from other receptors in ASER neurons

GCY family proteins are guanylyl cyclases that are expressed in different neurons in *C. elegans*. Owing to the sensor functions of GCY family proteins, *C. elegans* is more sensitive to the environment than other organisms are. Changes in oxygen levels (26), alkalinity (27), and carbon dioxide (28) in the environment activate GCY proteins in specific neurons. In addition, GCYs are involved in the aging process and lifespan of *C. elegans* (29, 30). GCY-5 is expressed in ASER chemosensory neurons. Several GCY proteins in ASER neurons sense different salt ions (31). GCY-5 has been reported to regulate the SOD-1 antagonistically with NPR-1 (17). However, the sensory function of GCY-5 and the mechanisms underlying its functions are unknown. According to our results, some special components in 4EM activated *GCY-5* and regulated the downstream signaling pathway (Fig. 1, *J* and *M*–*P*). To identify the ligand of *GCY-5* in 4EM, we performed nontargeted metabolomics analysis of 4EM and 20EM. We identified 97 differentially abundant components in 4EM (Fig. S5, *A–D* and Table S1). Most of the differentially abundant components in 4EM were matched to lipid molecules and organic acids in both positive- and negative-polarity modes. All these differential abundant components were related to amino acid metabolism and fatty acid metabolism pathways according to KEGG analysis, including “propanoate metabolism”, “glycine, serine and threonine metabolism”, and “phenylalanine, tyrosine and tryptophan biosynthesis” pathways (Fig. S5, *C* and *D*). These results suggest that *GCY-5* could be a receptor of specific metabolites associated with these metabolic pathways. In all, 97 components were identified as upregulated in 4EM. Of those, six were unidentified, eight were peptides or proteins, and 49 were not available through purchase (Table S1). For the remaining 34 ingredients, we investigated the fluorescence integrity of *pGCY-5::GCY-5::GFP/gcy-5* in irradiated worms after treatment. L-asparagine, birinapant, febuxostat, glycerol 3-phosphate, and indole were found to effectively promote the expression of *GCY-5* after irradiation (Fig. S5*F*). Furthermore, we confirmed the mRNA expression of *GCY-5* after different treatments. The results indicated that the expression of *GCY-5* was promoted by the ingredients screened above (Fig. S5*E*).

### Lysosome-related organelles formation might involve in *FAT-5*-mediated fat storage in lysosomes

Lysosomes are very important organelles for maintaining cell homeostasis. The function of lysosomes is very closely associated with lifespan. Lysosomes are modulated by multiple longevity pathways and directly lead to the extension of lifespan (11). Some drugs, such as metformin, promote longevity through the lysosomal pathway (32). On the other hand, lipid metabolism is also closely related to aging. The regulatory mechanism between fatty acids and lysosomes involves the regulation of lipid metabolism *via* the autophagy pathway (33, 34). However, there have been no studies on the effects of *FAT-5/SCD5* suppression on the lysosomal pathway. Our work revealed that *FAT-5* suppressed the lysosomal pathway after 4EM treatment and irradiation. In addition, we further investigated the regulatory effect of lysosomes on *FAT-5* (Fig. 4*E*). We found that *FAT-5*-mediated lipid storage in lysosomes might be regulated in a unidirectional manner. According to the results shown in Figure 4, *F*–*I*, the transcript levels of lysosome-related genes were significantly greater than those in WT cells (Fig. S4, *B*–*E*). Thus, we speculated that under normal conditions, the lysosomal pathway and the *FAT-5*-mediated fatty acid metabolism pathway are in balance and that gamma radiation or *GCY-5* induction disrupts this balance, affecting the longevity of *C. elegans* in another way. Previous work revealed that lysosome-related organelles (LROs) could be a special means of lipid storage under some unknown specific conditions (35, 36). In our work, lipids accumulated in lysosomes after treatment with 4EM after irradiation. Therefore, we speculated that LROs constitutes an acute mechanism for lipid storage under conditions of 4EM-induced protection from gamma radiation.

### *SCD5* might be a potential therapeutic target for radiation-induced injury

To facilitate the clinical application of the *SCD5*-mediated pathway, we examined the effects of *SCD5* in patients with various tumor types following radiotherapy (Fig. S6, *A*–*G*). KIRC was determined to be significantly influenced by SCD5. Consequently, we examined the *SCD5*-mediated pathway in 293T cells to investigate its therapeutic effects in normal tissues. The results presented in Figure 6 suggest that *SCD5* may serve as a novel target for mitigating side effects induced by radiotherapy in patients with KIRC. Furthermore, we conducted an analysis of drug sensitivity within the TCGA database, correlating the data with the expression levels of *SCD5* across KIRC patients (Fig. S7). Several drugs were screened for the sensitivity in patients with varying levels of *SCD5* expression. The results showed that patients with high SCD5 expression were predicted to be more sensitive to drugs such as austocystin D and BCL-LZH-4 (top 10 are listed in Fig. S7). These findings can provide valuable guidance for the combination of clinical radiotherapy and chemotherapy for KIRC patients.

## Conclusion

This study revealed that the *GCY-5* mediated *FAT-5/SCD5-*related pathway plays a significant role in protection from radiation-induced injury. Specifically, *FAT-5/SCD5* modulates the localization of lipids within lysosomes, rather than facilitating their storage in the cytoplasm or their degradation. This pathway is also functional in the human 293T cells. Bioinformatic analysis revealed that the expression level of SCD5 might affect the prognosis of KIRC patients after radiotherapy. Furthermore, the identification of the active components of 4EM, along with the development of a novel therapeutic strategy informed by bioinformatic analysis, represents significant avenues for future research. These efforts are expected to yield new insights into radiation protection and innovative approaches to tumor treatment.

## Experimental procedure

### Strains and reagents

The following strains of *C. elegans* were used: AB1: wild type; RB1000: gcy-5(ok921) *II*; PHX4456: *GCY-5(ok921) II; sybIs [pGCY-5-GCY-5-GFP-unc-54 3′UTR, pCFJ104*]; BX107: fat-5(tm420) *V*; BX150: *waEx18 [fat-5::GFP + lin15(+)]*; JJ1271: *glo-1(zu391)*. AB1, RB1000, BX107 and JJ1271 strains were obtained from the *Caenorhabditis Genetics Center* (CGC). BX150 was a kind gift from Prof. Chang Ming of Jiangnan University. The PHX4456 *C. elegans* allele was generated by SunyBiotech.

Drugs for screening were purchased from reagent companies. The concentrations, brands, and catalog numbers are listed in Table S1.

### Inactivation treatment for *Escherichia coli* OP50

*E. coli* OP50 serves as the primary nutritional source for *C. elegans*. In this study, both the experimental groups of worms and their respective control groups, which included variations in temperature, metabolites, and compounds, were provided with inactivated OP50. Initially, live OP50 bacteria were cultured in Luria-Bertani (LB) liquid medium within a shaking incubator set at 37 °C until the optical density at 600 nm (OD600) reached a range of 0.6 to 0.8. Subsequently, the cultured bacteria were subjected to inactivation by incubation at 65 °C overnight. From the point of hatching, the experimental worms were consistently fed with inactivated OP50 to ensure the absence of viable OP50 bacteria in the environment throughout the temperature or metabolite treatment phases.

### Lifespan assays

Unless otherwise indicated, lifespan studies were conducted at 20 °C. For the 4 °C treatment or EM treatment, worms in the L1 stage were exposed to 4 °C or EM for 4 h. After treatment, the worms were irradiated at 60 Gy using a Gammacell 40 Exactor (0.84 Gy/min, Atomic Energy of Canada Lim). The day after the worms hatched was considered day 1. For each lifespan assay, more than 90 worms were selected and transferred to fresh NGM every day throughout the spawning period. After the spawning period, the nematodes were transferred to fresh medium every 2 days. If the worms crawled off the plate, they were excluded from the analysis. Images were generated with GraphPad Prism 5 (GraphPad Software, Inc.).

### Extract medium preparation

To obtain medium from *C. elegans* at different temperatures, worms were first treated at 20 °C. After the worms reached the L1∼L2 stage, they were transferred to liquid NGM containing inactivated OP50 and divided into two groups: the 20EM and 4EM groups. *C. elegans* in the 20EM and 4EM groups were treated at 20 °C and 4 °C, respectively, for 4 h. The supernatant was subsequently collected from each group and sterilized with 0.22-μm filters. After ultracentrifugation at 100,000*g* for 30 min to remove the exosomes, the prepared EM was used immediately or kept at −80 °C for further use.

### Fluorescence staining and confocal analysis

To assess apoptosis in *C. elegans* after irradiation, worms in the L1 stage were treated as indicated and exposed to gamma radiation at 60 Gy. Twenty-four hours after irradiation, the worms in the different groups were collected and washed twice with M9 buffer. The worms were stained with TUNEL fluorescence staining solution for 1 h at 37 °C following the instructions of the TUNEL kit (#T2190, Solarbio). After the staining solution was washed away 2 to 3 times with M9 buffer, the worms were cultured with anesthetic (5 μM levamisole hydrochloride in M9 buffer, #B326BA1193, Sangon Biotech) and stained for further observation *via* confocal microscopy.

To investigate lipid distribution by fluorescence staining, LysoTracker Red (#C1046, Beyotime) and BODIPY (#D3922, Invitrogen) were used. Worms in the L1 stage were treated as indicated and exposed to gamma radiation at 60 Gy. Twenty-four hours after irradiation, the worms were collected and washed twice with M9 buffer. LysoTracker Red and BODIPY were diluted as indicated by the manufacturer's instructions. The worms were stained with LysoTracker Red and BODIPY working solutions for 30 min at 37 °C following the manufacturer's instructions. After the staining solutions were washed away 2 to 3 times with M9 buffer, the worms were cultured with anesthetic and shaded for further observation by confocal microscopy.

Fluorescence staining in lipid distribution observation was visualized by confocal microscopy (Leica, TCS SP8). Fluorescence staining in lysosomal function observation (LAMP2 and cathepsin B staining) was visualized by confocal microscopy (Zeiss, LSM980). The LysoTracker signal was observed at emission wavelengths of 570∼610 nm with excitation at 552 nm. The BODIPY signal was observed at emission wavelengths of 500∼545 nm with excitation at 488 nm. The TUNEL signal was observed at emission wavelengths of 570∼610 nm with excitation at 552 nm.

For ROS staining, intestines were isolated following the protocol described by Jessica L Hill, *et al.* (37). Tissues were stained with DCFH-DA (#S0033S, Beyotime) and Hoechst 33,342 (#C1022, Beyotime). The ROS signal was observed at wavelengths of 500∼545 nm with excitations at 488 nm. The Hoechst signals were observed at emission wavelengths of 450∼470 nm with excitation at 408 nm.

### Immunofluorescence assay

Dispense 20 to 100 μl of 0.2 mM levamisole/PBST onto a concave glass slide, and subsequently place 10 to 20 nematodes on the slide. Utilize a 1 ml syringe to dissect the nematodes, thereby exposing their intestines. Transfer 10 μl of the resultant sample to a 1.5 ml tube. Continue this procedure until a total of 100 to 200 nematodes have been collected. Following fixation with formaldehyde, introduce 600 μl of Quench solution (1% formamide, 6% H_2_O_2_, and 0.5% Triton X-100 in 1 × PBS) and expose the sample to intense light for a duration of 2 h. Rinse the sample twice with PBST, each rinse lasting 5 min. Subsequently, add 400 μl of PBST/1% BAS and incubate at room temperature for 1 h to facilitate blocking. Introduce 150 to 200 μl of the primary antibody, diluted with PBST/1% BAS, and incubate at room temperature for 2 h or alternatively, overnight at 4 °C. The samples were washed with PBST four times, with each wash lasting 5 min. Subsequently, an equivalent volume of secondary antibody was added and incubated in the dark for 1 h. Following this, the samples were washed with PBST four additional times. A 20 μl aliquot of Hoechst staining solution was then applied in the dark for 5 to 10 min. The samples were subsequently transferred to a 2% agarose pad for mounting. The LAMP2 (#A22216, Abclonal) signal was observed at wavelengths of 500∼545 nm with excitations at 488 nm excitation. The primary antibody against cathepsin B (#A0967, Abclonal) was conjugated with an Alexa Fluor 647-labeled secondary antibody (ab150075, Abcam). Cathepsin B signal was observed at wavelengths of 660∼670 nm with excitations at 652 nm.

### Real-time PCR

Worms in the L1 stage were exposed to different temperatures or EM for 4 h. After the indicated treatment, the worms were irradiated at 60 Gy. Twenty-four hours after irradiation, the worms were collected and washed twice with M9 buffer. After the supernatant was removed, the worms were resuspended in 1 ml of TRIzol (RNAiso plus, #9109) and disrupted *via* ultrasonication (950E, Ningbo Scientz Biotechnology Co., Ltd) to extract total RNA. The following procedures were performed according to a standard laboratory RNA extraction protocol. Total RNA was reverse transcribed with a reverse transcription kit (#RR037).

Real-time PCR was used to quantify the relative expression of genes. *ACT-1* was used as the reference gene for all real-time PCR assays performed in this work. We used SYBR Green Mix (ROX) (#4913914001, Roche, Switzerland) as the fluorescent dye. The primers used in this work are listed in Table S1.

### Cell culture and transfection

The 293T cell lines were initially procured from the ATCC. Prior to utilization, it is essential to conduct *mycoplasma* testing using Hoechst staining. 293T cells were cultured in Dulbecco's modified Eagle's medium (DMEM) supplemented with 10% fetal bovine serum (#C04001, Vivacell), penicillin, and streptomycin (#PB180120, Pricella) in a humidified incubator at 37 °C with an atmosphere containing 5% carbon dioxide. The cells were routinely passaged every 2 to 3 days. To overexpress SCD5, an SCD5 expression plasmid was constructed with the pcDNA3.1 vector. Next, the constructed plasmid was transiently transfected into 293T cells *via* Lipofectamine 3000 (#L3000015, Invitrogen)

### Western blot

Nematodes in the L1 stage were exposed to different temperatures or extraction medium for 4 h. Nematodes were irradiated at 60 Gy after treatment, collected 24 h later and washed twice with 1 × M9 buffer. After centrifugation, the pellet was resuspended in RIPA lysis buffer (Solarbio, #R0010). Total proteins were extracted *via* ultrasonication (Model 950E; Ningbo Scientz Biotechnology Co., Ltd). The proteins were subsequently separated *via* SDS-PAGE and transferred onto PVDF membranes. After blocking in 5% non-fat milk powder, the membrane were incubated with antibodies against VHA-12/ATP6V1B2 (1:2000, Proteintech), SPE-5/ATP6V1B1 (1:5000, Proteintech), GLO-1/RAB32 (1:500, Proteintech), β-actin (1:20,000, Proteintech), and SCD5 (1:500, ABclonal) overnight at 4 °C. The membranes were then washed and incubated with a secondary antibody for 45 min. Image detection was performed using a Bio-Rad imaging system. The gray value of each band was measured *via* Image J. In determining the gray value of the target protein, it is essential to concurrently calculate the gray value of β-actin present on the same film. The data presented in this study are expressed as the ratio of the target protein to β-actin.

### Flow cytometry analysis

The detection of apoptosis was performed using an Annexin V-FITC apoptosis detection kit (#C1062S, Beyotime). The vector control and SCD5-OX cells were seeded into 6-well plates at a confluence of 40%. The cells were irradiated at 6 Gy. After 24 h, the cells were collected and suspended in a binding buffer containing Annexin V-FITC. The cell suspension was further incubated with propidium iodide (PI) for 15 min. Apoptosis was detected using by a BD FACS Verse flow cytometer (BD Biosciences) and quantitatively analyzed *via* Flow Jo software.

### Transmission electron microscopy

Samples were prepared in accordance with previously established methods (38). For the high-pressure freezing (HPF) process, multiple live *C. elegans* samples were placed into Type A specimen carriers with a 100-μm well, containing 20% bovine serum albumin (BSA) to prevent the formation of voids and overfilling. The carriers were promptly sealed with sapphire discs and subjected to freezing *via* an HPF system (Compact-03, Switzerland). Post-HPF, the frozen samples were immersed in a freezing tube containing 1% osmium tetroxide in 100% acetone and transferred to a freeze substitution (FS) device (Leica EM AFS2) under the following conditions: T1 = −90 °C for 72 h, S1 = 2 °C/h, T2 = −60 °C for 12 h, S2 = 3 °C/h, T3 = −20 °C for 10 h, followed by a gradual warming to 4 °C at a rate of 5 °C/h. The samples were subsequently subjected to three rinses in 100% acetone, each lasting 15 min at 4 °C, followed by a single rinse at room temperature (RT). The samples were then stained with 1% uranyl acetate dissolved in a 90% acetone/10% methanol solution (filtered prior to use) for 2 h in the dark at RT. Following staining, the samples were rinsed four times in 100% acetone, each rinse lasting 15 min at RT. The samples were subsequently infiltrated with a graded series of resin and acetone mixtures (1:3, 1:1, and 3:1). The resin was composed of 16.2 ml of SPI-PON812, 10 ml of dodecenyl succinic anhydride (DDSA), 8.9 ml of nadic methyl anhydride (NMA), and 1.5% benzyl dimethyl aniline (BDMA). The samples were subsequently transferred to 100% resin, which was replaced three times over the subsequent 2 days while on a rotator. The samples were ultimatetly embedded in fresh resin and subjected to polymerization for 12 h at 45 °C, followed by an additional 48 h at 60 °C. Ultrathin sections, measuring 70 nm in thickness, were prepared using an ultramicrotome (Leica EM UC6). These sections were subsequently imaged with a transmission electron microscope (FEI Tecnai Spirit 120 kV, FEI Company) equipped with an EMSIS CCD camera (VELETA, EMSIS).

### Transcriptome analysis

Transcriptome analysis was performed by Novogene Co., Ltd. The main methods were as follows. RNA was extracted from *C. elegans* following the indicated treatments using TRIzol, and 3 μg of RNA per sample was used for RNA sample preparation. Sequencing libraries were generated using the NEBNext Ultra RNA Library Prep Kit for Illumina (NEB) following the manufacturer's recommendations, and index codes were added to attribute sequences to each sample.

Clustering of the index-coded samples was performed on a cBot Cluster Generation System using the TruSeq PE Cluster Kit v3-cBot-HS (Illumina) according to the manufacturer's instructions. After cluster generation, the library preparations were sequenced on an Illumina HiSeq platform, and 125 bp/150 bp paired-end reads were generated.

Differential expression analysis between two conditions/groups (two biological replicates per condition) was performed *via* the DESeq2 R package (1.16.1). DESeq2 provides statistical routines for assessing differential expression in digital gene expression data using a model based on the negative binomial distribution. The resulting *p* values were adjusted *via* Benjamini and Hochberg's approach for controlling the false discovery rate. Genes with an adjusted *p* value < 0.05 according to DESeq2 were considered differentially expressed.

### Nontarget metabolomics analysis

Transcriptome analysis was completed by Novogene Co., Ltd. The main methods were as follows. Media were isolated from worms *via* centrifugation at 5000 rpm for 10 min. The exosomes were removed by ultracentrifugation at 100,000*g* for 30 min. The EM was diluted to a 60% final concentration with methanol. The EM samples were filtered with a 0.22-μm filter and then were centrifuged at 15,000*g*, 4 °C for 10 min. The samples were injected onto a Hyperil Gold column (100 × 2.1 mm, 1.9 μm) *via* a 16-min linear gradient at a flow rate of 0.2 ml/min. The eluents for the positive-polarity mode were 0.1% FA in water as eluent A and methanol as eluent B. The eluents for the negative-polarity mode were 5 mM ammonium acetate (pH 9.0) as eluent A and methanol as eluent B.

The raw data files generated by UHPLC-MS/MS were processed *via* the Compound Discoverer 3.1 (CD3.1, Thermo Fisher) to perform peak alignment, peak selection, and quantitation for each metabolite. Peaks were matched with the mzCloud (https://www.mzcloud.org/) and ChemSpider (http://www.chemspider.com/) databases to obtain the accurate qualitative and relative quantitative results. Statistical analyses were performed *via* the statistical software R (R version R-3.4.3), Python (Python 2.7.6 version) and CentOS (CentOS release 6.6), When the data were not normally distributed, normal transformations were attempted *via* the area normalization method.

### Nontargeted lipidomic analysis

Nontargeted lipidomic analysis was performed by APTBIO Co., Ltd. The methods are summarized below. Lipids were extracted according to the MTBE method. Briefly, 200 μl of water was added to the sample and vortexed for 5 s. Subsequently, 240 μl of precooled methanol was added, and the mixture was vortexed for 30 s. Then, 800 μl of MTBE was added, and the mixture was ultrasonicated for 20 min at 4 °C, followed by incubation for 30 min at room temperature. The mixture was subsequently centrifuged at 14,000*g* for 15 min at 10 °C, and the upper organic solvent layer was obtained and dried under nitrogen. Reverse-phase chromatography was applied for LC separation *via* a CSH C18 column (1.7 μm, 2.1 mm × 100 mm, Waters). The lipid extracts were redissolved in 200 μl of 90% isopropanol/acetonitrile and centrifuged at 14,000*g* for 15 min; finally, 3 μl of sample was injected. Solvent A was acetonitrile–water (6:4, v/v) with 0.1% formic acid and 0.1 mM ammonium formate, and solvent B was acetonitrile–isopropanol (1:9, v/v) with 0.1% formic acid and 0.1 mM ammonium formate. The initial mobile phase was 30% solvent B at a flow rate of 300 μl/min. These conditions were held for 2 min followed by a linear increase to 100% solvent B in 23 min and equilibration at 5% solvent B for 10 min.

### RNA-Seq data access

The RNA expression matrix and clinical information of kidney renal clear cell carcinoma (KIRC) patients from TCGA were used for survival analysis and drug sensitivity prediction analysis. Expression data were downloaded *via* the TCGA biolinks (39) package (version 2.32.0), and clinical information was downloaded from the UCSC Xena browser (https://xenabrowser.net/datapages/). A total of 533 transcriptome analysis samples were downloaded, of which 528 had corresponding clinical information, including radiotherapy and survival time.

### Survival analysis

The study cohort was stratified through maximally selected rank statistical analysis implemented *via* the surv_cutpoint tool in the survminer package (version 0.4.9, https://github.com/kassambara/survminer). Patient survival patterns were visualized through Kaplan‒Meier estimation curves, with between-group differences assessed *via* the log-rank test. To evaluate prognostic correlations, survival outcomes were quantitatively analyzed against clinical parameters through univariable Cox regression modeling. A *p* value less than 0.05 was considered statistically significant.

### Drug sensitivity prediction analysis

The OncoPredict (version 1.2) (40) package was used for drug sensitivity prediction analysis of TCGA KIRC data. The reference training data were downloaded from the CTRP v2 database (41). The training and testing data matrices were homogenized *via* the eb method, and genes with less than 20% variation were removed from the testing data. The top 10 drugs with high predicted sensitivity and strong predicted correlations with SCD5 were identified. The *p* value for the IC50 comparison was calculated *via* Student's *t* test, whereas the *p* value and regression coefficient for the correlation analysis between the IC50 and SCD5 level were calculated *via* smooth linear regression. A *p* value less than 0.05 was considered statistically significant.

### Statistical analysis

Each experiment was repeated at least three times. Mean values from the two groups were compared *via* Student's *t* test. Transcriptome sequencing results were assessed *via* Tukey's HSD test, and two-way ANOVA was used to compare the results. All statistical analyses were performed *via* SPSS 17.0 software. *p* < 0.05 was considered statistically significant.

## Data availability

The transcriptome sequencing datasets and lipidome data have been submitted to the National Genomics Data Center (NGDC), China National Center for Bioinformation (CNCB), and are accessible *via* the following link: https://ngdc.cncb.ac.cn/(Project ID: PRJCA043624). All research data generated or analyzed in the course of this study are incorporated within this published article and its Supplementary Material.

## Supporting information

This article contains supporting information.

## Conflict of interest

The authors declare that they have no conflicts of interest with the contents of this article.
