# Supplementary material for: FAT-5/SCD5-mediated lipid localization in lysosomes alleviates gamma radiation injury in Caenorhabditis elegans
Source: J Biol Chem. 2025 Sep 2;301(10):110674. doi: 10.1016/j.jbc.2025.110674 (PMC12509980; doi:10.1016/j.jbc.2025.110674)
Supplement: Supporting Information [file mmc1.docx]

**Supporting Information**


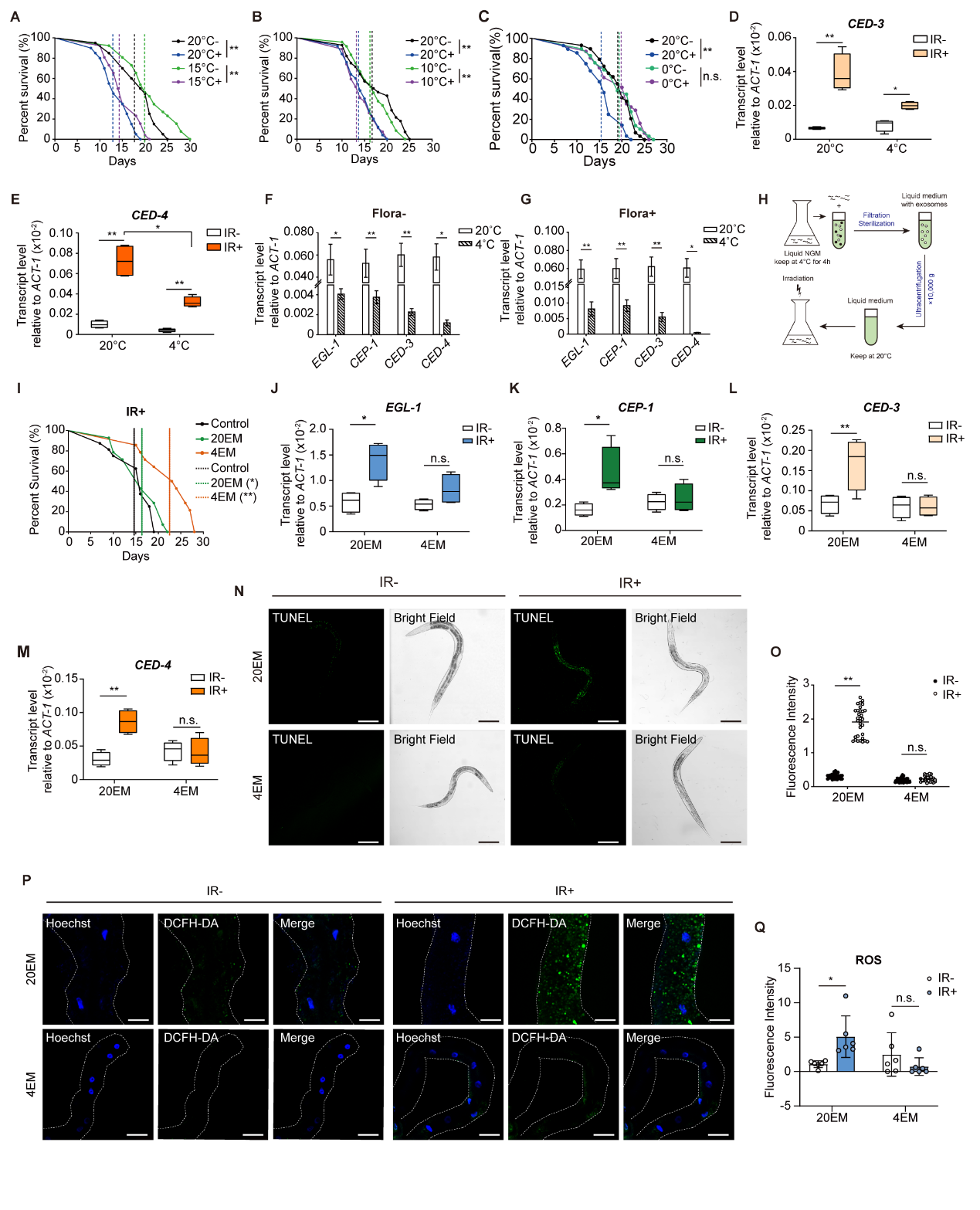


**Figure S1. Extraction medium incubated at 4 °C mitigated irradiation-induced injury.** (A)-(C) Kaplan–Meier survival curves of WT worms after exposure to 15 °C (A), 10 °C (B) or 0 °C (C) for 4 hours with or without irradiation. The dotted lines indicate the average lifespan of each group. **, P<0.01; n.s., no significant. (D)-(E) Relative transcript levels of *CED-3* and *CED-4* in worms cultured at 20 °C or 4 °C. At least 3 replicates were included for each group. (F)-(G) Relative transcript levels of *EGL-1*, *CEP-1*, *CED-3* and *CED-4* in worms cultured in media supplemented with (F) or without (G) microorganisms. At least 3 replicates were included for each group. (H) Scheme of the extraction medium treatment protocol. Worms in the L1~L2 stage were cultivated in liquid NGM at 20 °C or 4 °C for 4 hours. The worms were removed from the medium via centrifugation. The medium was sterilized with a 0.22-μm filter to remove microorganisms. EM was applied to the recipient worms for 4 hours, after which the worms were irradiated at 60 Gy. (I) Kaplan–Meier survival curves of 20EM- or 4EM-treated *C. elegans* after irradiation. Worms were selected for survival curve analysis after different treatments. Dead worms were counted every day. The dotted lines indicate the average lifespan of each group. (J)-(M) Relative transcript levels of *EGL-1* (J), *CEP-1* (K) , *CED-3* (L), and *CED-4* (M) in the different groups. At least 3 replicates were included for each group. (N)-(O) TUNEL staining after irradiation of worms treated with 20EM or 4EM (N) and the results of statistical analysis (O). Scale bar, 100 μm. (P) ROS accumulation in the intestines of worms in each group. Scale bar, 25 μm. (Q) Fluorescence intensity of the data in (N). For each group, more than 6 images were captured and analyzed. Individual data points for worms from each group are shown in the graph. In (A)-(O), *, P<0.05, **, P<0.01; n.s., not significant.


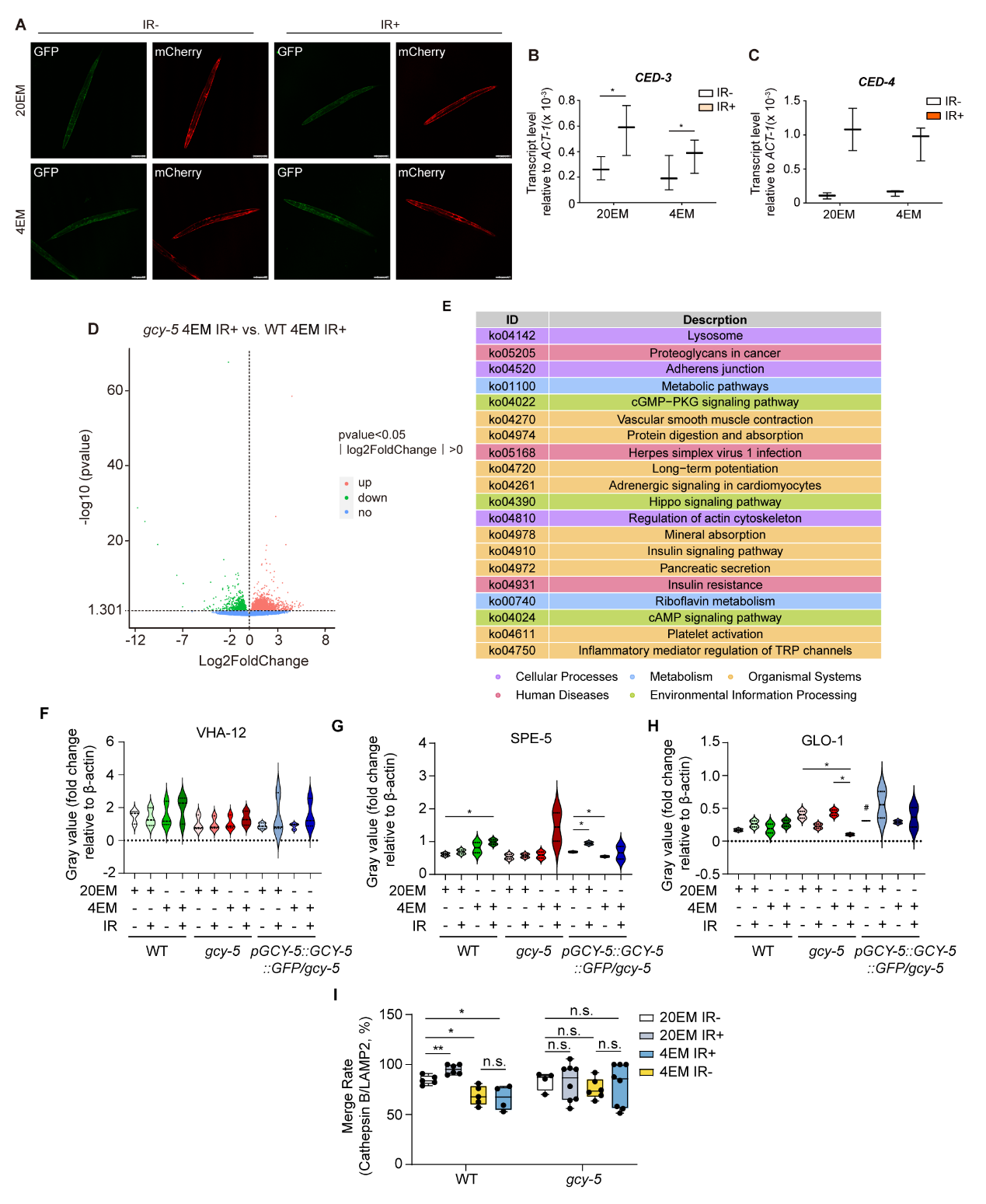


**Figure S2. 4EM mitigates radiation-induced injury through GCY-5.** (A) Images of *pGCY-5::GCY-5::GFP/gcy-5* in different groups. mCherry indicates the reference expression. GFP indicates GCY-5 expression. Scale bar, 100 μm. (B-C) Relative transcript levels of CED-3 and CED-4 in *gcy-5* mutant worms treated with 20EM and 4EM. At least 3 replicates were included for each group. *, P<0.05. (D) Volcano plot of the transcriptomic analysis between the *gcy-5* 4EM IR+ and WT 4EM IR+ groups. Green dots indicate downregulated genes. Red dots indicate upregulated genes. (E) Kyoto Encyclopedia of Genes and Genomes (KEGG) analysis of the genes with differential expression between the *gcy-5* 4EM IR+ and WT 4EM IR+ groups, as an annotation to Figure 2A with the full name of each pathway. (F-H) Gray values of proteins in Figure 2C. The gray value of each band, including that of β-actin on the same film, was measured via Image J. The data shown in this graph represent the fold change in protein expression to that of β-actin. These results were obtained from three Western blot experiments. *, P<0.05; #, P<0.05, compared with WT; not significant data are not labeled in graph. (I) Statistical analysis of the data in Figure 3H. The fluorescence intensity in WT (F) and *gcy-5*-deficient (G) worms was determined in each stained image. For each group, over 30 images were counted and organized. Individual data points for worms from each group are shown in the graph. *, P<0.05; **, P<0.01; n.s., not significant.


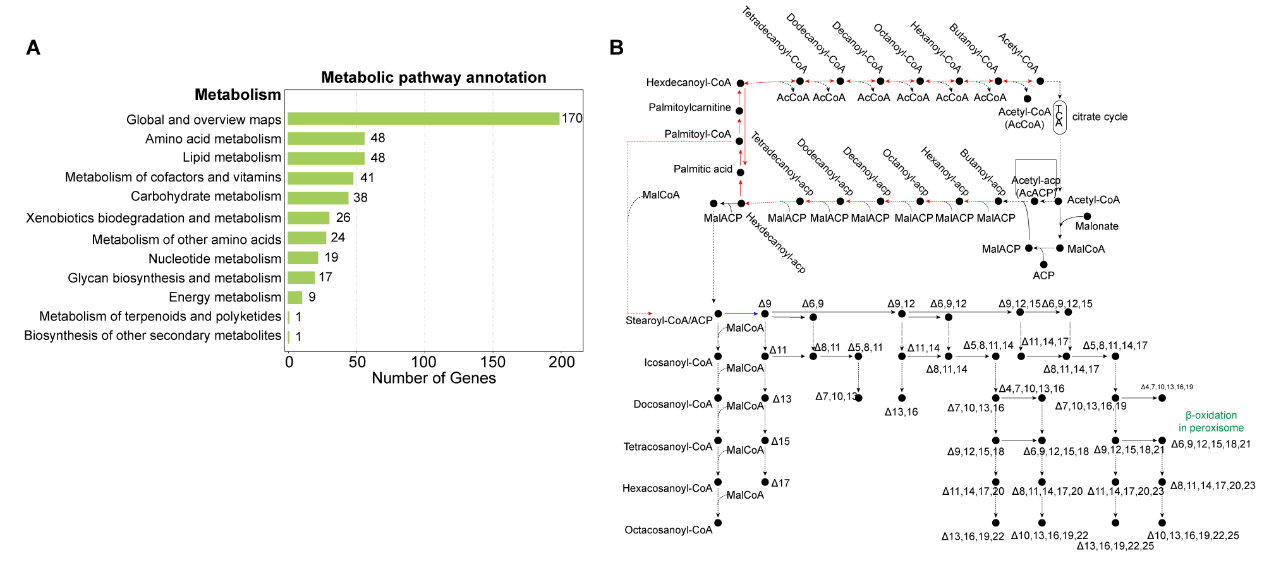


**Figure S3. Lipid metabolism is involved in the *GCY-5* mediated mechanism.** (A) The genes screened in the ‘*gcy-5* IR+ vs. WT IR+’ comparison belong to the metabolism category. (B) Diagram of the whole pathway of fatty acid metabolism. The red arrows indicate genes whose expression was upregulated in the transcriptome analysis. The blue arrow indicates the process involving only the downregulated gene, *FAT-5*.


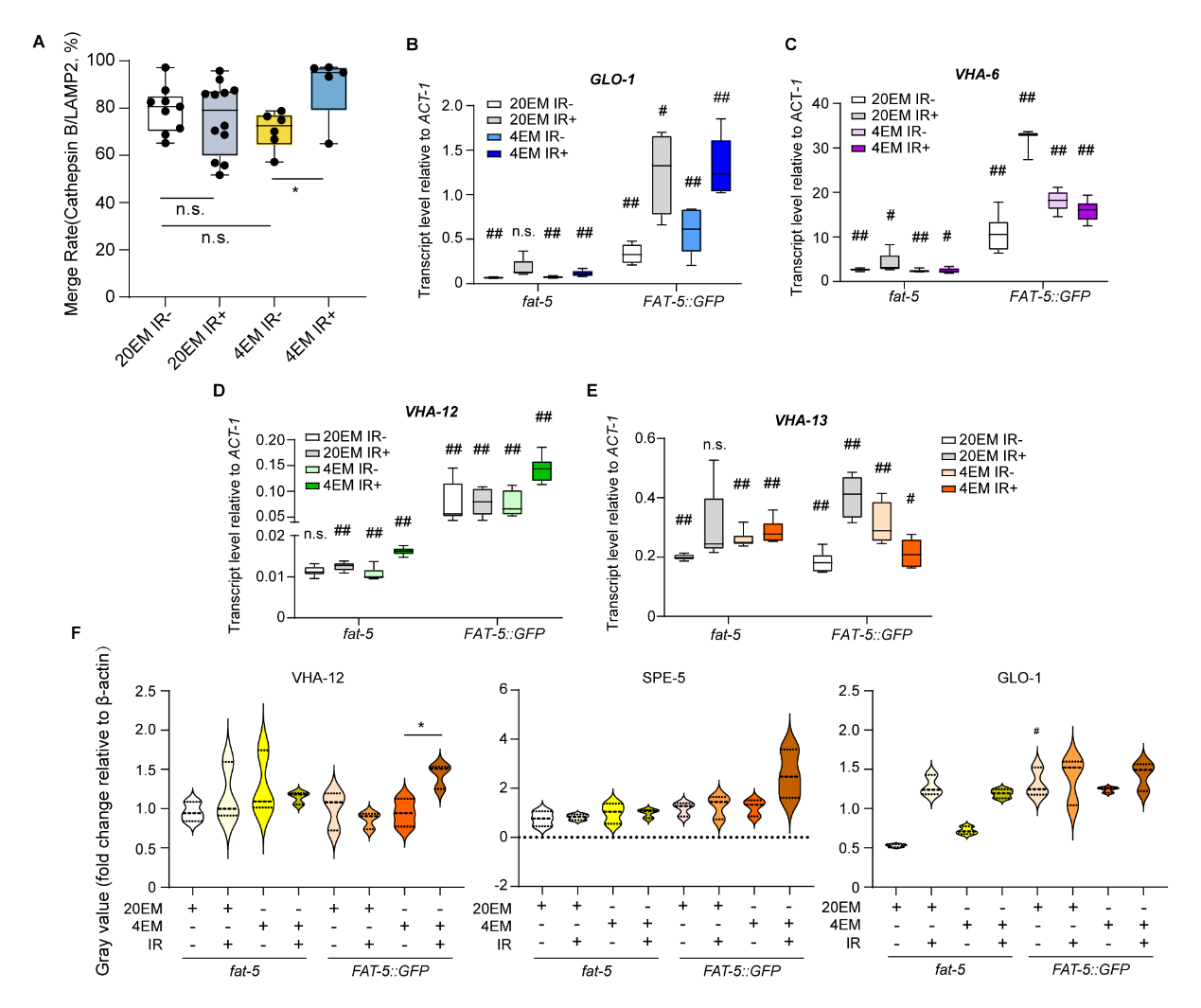


**Figure S4. *Fat-5*-mediated fatty acid metabolism is involved in the *GCY-5*-mediated pathway.** (A) Statistical analysis of the data in Figure 4E. The fluorescence intensity in *fat-5* mutant worms was determined in each stained image. For each group, over 30 images were counted and organized. Individual data points for worms from each group are shown in the graph. *, P<0.05; **, P<0.01; n.s., not significant. (B)-(E) Fold changes in the expression of GLO-1 (B), VHA-6 (C), VHA-12 (D), VHA-13 (E) after 4EM treatment in *fat-5* and *FAT-5*::GFP *C. elegans*. At least 3 replicates were included for each group. More than 1000 worms were collected from each sample. #, P<0.05, ##, P<0.01; n.s., not significant, compared with WT. (F) Gray values of each proteins in Figure 4J. The gray value of each band, including that of β-actin on the same film, was measured via Image J. The data shown in this graph represent the fold change in protein expression relative to that of β-actin. These results were obtained from three Western blot experiments.


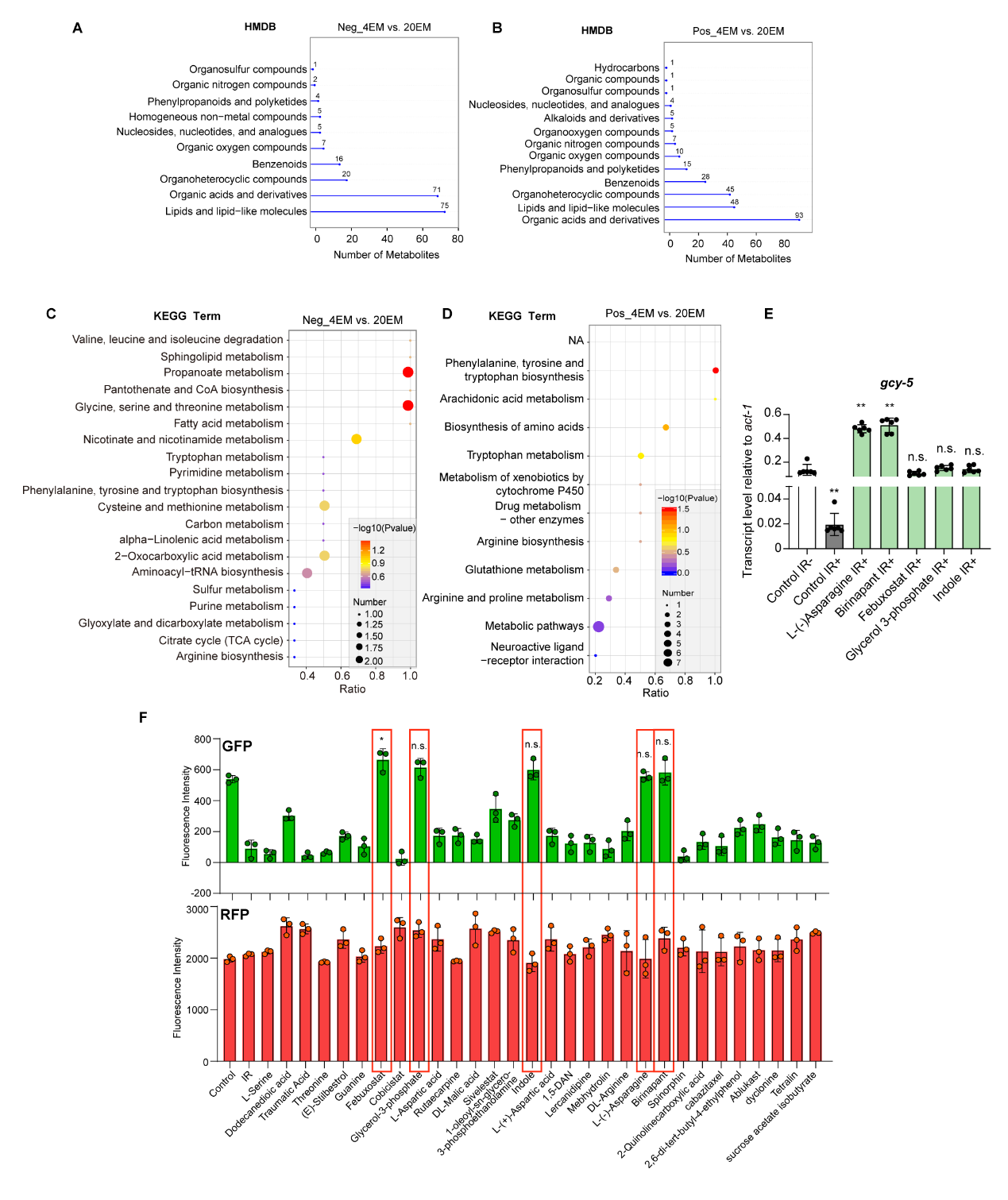


**Figure S5. Active ingredient screening by nontargeted metabolomic analysis.** (A)-(B) Metabolite classification in negative-polarity (A) and positive-polarity (B) modes according to the HMDB. The numbers in the graph refer to the number of metabolites enriched in each item. (C)-(D) Kyoto Encyclopedia of Genes and Genomes (KEGG) analysis of the differentially abundant metabolites between 4EM and 20EM in negative-polarity (C) and positive-polarity (D) modes. (E) Relative expression of *GCY-5* in irradiated WT worms after different treatments. At least 3 replicates were included for each group. More than 1000 worms were collected from each sample. *, P<0.05; **, P<0.01; n.s., not significant. (F) Fluorescence intensity of GFP (upper) and RFP (lower) in *pGCY-5::GCY-5::GFP/gcy-5* after different drugs treatments. A black 96-well plate was used in this experiment. There were 50 worms in each well. The vertical ordinate represents the fluorescence intensity of 10 worms in different groups. At least 3 replicates were included in each group. *, P<0.05; **, P<0.01; n.s., not significant.


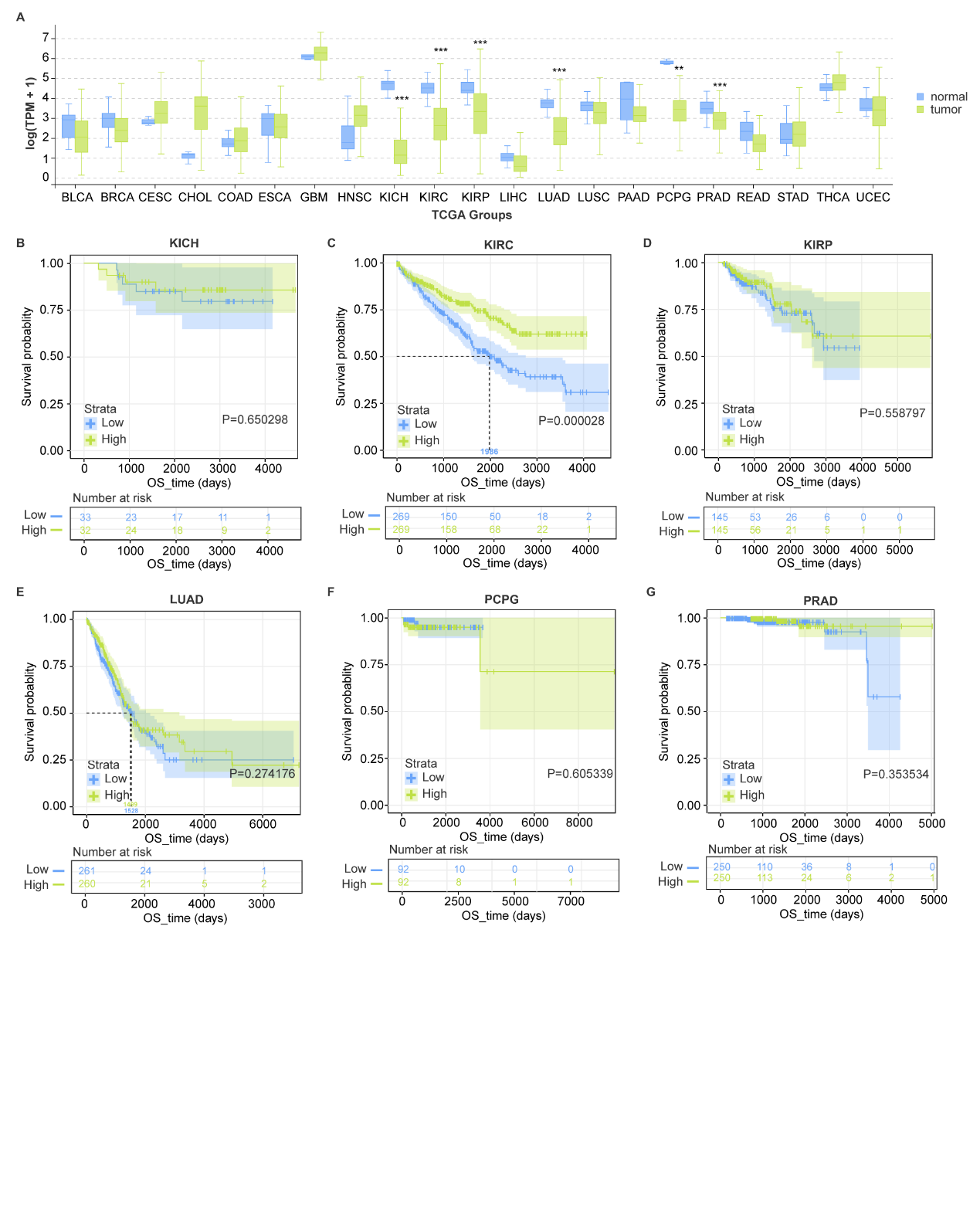


**Figure S6. Elevated expression of *SCD5* is associated with a reduced survival rate in patients with KIRC.** (A) Expression levels of *SCD5* in various tumor types relative to that in corresponding paracarcinoma tissues. **, P<0.01; ***, P<0.001. (B-G) Survival rates of 6 tumor types with significantly different *SCD5* expression, namely, KICH (B), KIRC (C), KIRP (D), LUAD (E), PCPG (F), and PRAD (G).


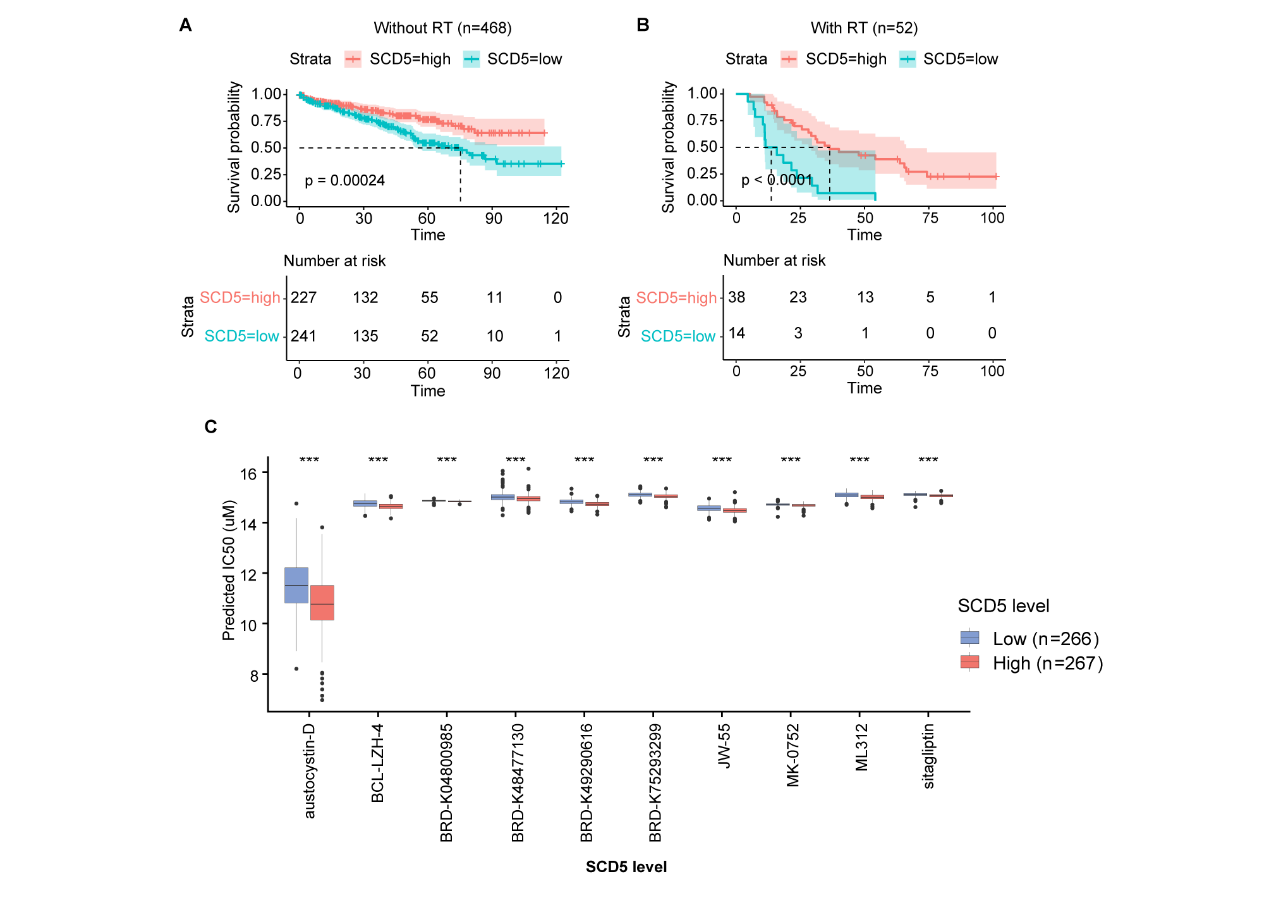


**Figure S7. The expression of SCD5 influences the prognosis of radiotherapy in patients with KIRC.** (A)-(B) Survival probability of patients with KIRC in relation to the expression levels of *SCD5*, stratified by the administration of radiotherapy. The datasets utilized were sourced from the public available TCGA database. The cohort comprises 468 patients who received radiotherapy (A) and 52 patients who did not receive radiotherapy (B). (C) Drug sensitivity analysis within the TCGA database revealed a correlation with the expression levels of SCD5 in KIRC. The top ten drugs are shown. ***P<0.001.
